# Cisplatin/5-Fluorouracil (5-FU) Versus Carboplatin/Paclitaxel Chemoradiotherapy as Definitive or Pre-Operative Treatment of Esophageal Cancer

**DOI:** 10.7759/cureus.12574

**Published:** 2021-01-08

**Authors:** Cole Steber, Ryan T Hughes, Emory R McTyre, Michael Soike, Michael Farris, Beverly J Levine, Boris Pasche, Edward Levine, Arthur W Blackstock

**Affiliations:** 1 Radiation Oncology, Wake Forest School of Medicine, Winston-Salem, USA; 2 Radiation Oncology, Prisma Health Cancer Institute, Greenville, USA; 3 Radiation Oncology, University of Alabama at Birmingham School of Medicine, Birmingham, USA; 4 Epidemiology, Wake Forest School of Medicine, Winston-Salem, USA; 5 Hematology and Oncology, Wake Forest School of Medicine, Winston-Salem, USA; 6 Surgery Oncology, Wake Forest School of Medicine, Winston-Salem, USA

**Keywords:** esophageal cancer, chemotherapy, cisplatin/5-fu, carboplatin/paclitaxel, radiation therapy, trimodality therapy, definitive chemoradiation, locally advanced esophageal cancer

## Abstract

Purpose

To determine the efficacy and toxicity of two standard chemotherapy regimens used concurrent with radiation for the treatment of esophageal cancer: cisplatin/5-fluorouracil (5-FU) and carboplatin/paclitaxel.

Materials and methods

We prospectively reviewed records of 364 patients with histologically confirmed stage I to IVA esophageal cancer receiving chemoradiotherapy (CRT) with or without resection. All patients received surgical evaluation and imaging at presentation as well as following completion of their course of CRT. Treatment and prognostic variables were compared across the two chemotherapy regimens.

Results

We identified 261 patients treated with concurrent carboplatin/paclitaxel (n = 133) or cisplatin/5-FU (n = 128). Weight loss during CRT was lower in patients receiving carboplatin/paclitaxel (median: 7.0 pounds; 4.1% body weight) vs. cisplatin/5-FU (median: 11.0 pounds; 6.5% body weight) (p < 0.01). In 117 patients receiving trimodality therapy, post-operative death rates within one month of resection were similar. Pathologic complete response was better with carboplatin/paclitaxel vs. cisplatin/5-FU, 29.6% vs. 21.8% (p = 0.03), respectively. In the multivariable analysis, there was no association between chemotherapy regimen and overall survival (OS) or progression-free survival (PFS), though there was a trend toward improved OS with carboplatin/paclitaxel with a HR = 0.75 (p = 0.08). Further analysis revealed that trimodality therapy and stage were predictors for improved OS and PFS while female gender and grade predicted for improved PFS.

Conclusions

Carboplatin/paclitaxel was associated with decreased weight loss and improved pathologic response for trimodality patients when compared to cisplatin/5-FU. We observed no differences in OS, PFS, or post-operative death by chemotherapy regimen for both the entire cohort and trimodality patients.

## Introduction

Esophageal cancer remains a serious health problem, with 17,650 new cases for the year 2019 in the United States alone [1]. Due to its aggressive nature and significant metastatic potential, the five-year survival rate for all patients diagnosed rarely exceeds 40% and minimal improvement in survival has been achieved over the past 20 years [1-3].

Although there is currently no global consensus [4], preoperative treatment with chemotherapy or chemoradiotherapy (CRT) followed by esophagectomy is an accepted standard for managing locally advanced esophageal cancer within the United States [5] and Europe [6]. Randomized trials and meta-analyses have shown that multimodality therapy is superior to surgery alone, and preoperative therapy is superior to postoperative therapy [2,4,7-9]. While direct comparisons of chemotherapy versus CRT remain limited, there are several recent randomized studies suggesting higher pathologic complete response (pCR) rates and a lower frequency of residual lymph-node disease with CRT [10-12].

What has not been well studied, is the optimal neoadjuvant chemotherapy to use as part of a CRT regimen. There have been no randomized studies comparing the two most standard chemotherapy regimens for the treatment of esophageal cancer to date. The standard treatment had long been neoadjuvant chemoradiotherapy with cisplatin and 5-fluorouracil (5-FU), despite its significant grade 3 and 4 toxicity rate of 30%-57% [6,13-15]. The considerable morbidity attendant to the use of cisplatin/5-FU led to the evaluation of other regimens compatible with concomitant radiation. The addition of docetaxel to cisplatin/5-FU increased response rates and survival, however at the risk of increased toxicity [15-17]. Two recent randomized trials, including the CROSS trial, demonstrated neoadjuvant chemoradiotherapy with weekly carboplatin and paclitaxel with concurrent radiotherapy resulted in better toxicity profiles (grade ≥ 3 toxicity rates of 18% and 23%, respectively) [9,18]; however, the use of carboplatin/paclitaxel in the CROSS trial was based on a previous phase II study [19], and no direct comparisons between carboplatin/paclitaxel and cisplatin/5-FU were made.

Since the publication of the CROSS trial, carboplatin/paclitaxel has now become an accepted standard concurrent chemotherapy regimen. Given these initial reports indicating a less severe toxicity profile and similar efficacy with neoadjuvant carboplatin/paclitaxel, there is growing interest in determining to what extent concomitant use of cisplatin/5-FU differs from carboplatin/paclitaxel in terms of overall clinical outcomes. In this study we examine the largest cohort of patients reported which compares these two chemotherapy regimens to determine the influence of the different definitive CRT or preoperative regimens on treatment-related toxicities, response rates, complication rates, and overall outcomes. This article was previously presented in abstract form as a poster presentation at the American Society for Therapeutic Radiation and Oncology 59th Annual Meeting on September 24-27, 2017.

## Materials and methods

In an Institutional Review Board approved retrospective analysis of a prospectively maintained database, we reviewed the records of patients with histologically confirmed stage I to IVA esophageal cancer [20] receiving CRT with or without resection with curative intent at the Wake Forest University Comprehensive Cancer Center between June of 1999 and December of 2018. Clinicopathological and follow-up data were collected in a prospective database and analyzed. Eligibility criteria for patients included age ≥ 18 years, histologically confirmed esophageal cancer at presentation, and treatment with concurrent CRT with or without surgical resection.

All patients underwent initial pretreatment staging. This included evaluations from medical oncology, radiation oncology, and surgical oncology, blood count and liver function testing, an upper gastrointestinal endoscopy with histologic biopsy and endoscopic ultrasonography and computed tomography (CT) imaging. [18F]fluorodeoxyglucose positron emission tomography (FDG-PET) with computed tomography correlation was also performed as part of the pretreatment staging in all patients.

The choice of neoadjuvant chemotherapy was at the discretion of the medical oncologist and depended on the patient’s general condition and available evidence at the time of treatment. Different common chemotherapy regimens used throughout the 20-year duration of the study included cisplatin/5-FU, carboplatin/paclitaxel, carboplatin/5-FU, or capecitabine as a monotherapy. Radiotherapy was planned using a three-dimensional conformal technique with all patients and delivered with megavoltage linear accelerators to a median dose of 50.4 Gy.

All patients underwent surgical evaluation pre- and post-CRT, including both restaging CT scans post-CRT treatment and an evaluation of medical fitness to undergo surgery. Esophagectomy was generally performed four to eight weeks after CRT by transhiatal or transthoracic technique at the discretion of the treating surgeon according to the location of the primary tumor. Digestive tract continuity was restored using a gastric conduit reconstruction and gastric drainage with either a pyloroplasty or pyloromyotomy. Patients who did not undergo surgery were either medically or surgically inoperable or declined surgery for other reasons.

Imaging with or without pathological examination was used to determine response to treatment. Imaging consisted of restaging scans after the completion of CRT and before resection in those patients receiving trimodality therapy. Restaging scans consisted of either FDG-PET with computed tomography correlation or computed tomography (CT) alone. FDG-PET images were interpreted by an experienced nuclear radiologist. Quantitative analysis was performed using standardized uptake values (SUVs), which were calculated as the maximum value one hour after injection of 15 to 20 mCI of [18F]FDG. FDG-PET response criteria were defined before data analysis. As reported in our prior reports [21], FDG-PET complete response after CRT was defined as SUV ≤ 3 to allow for mild hypermetabolic activity, which may represent post-treatment esophagitis. FDG-PET partial response was defined as ≥30% reduction in both primary and nodal disease, while FDG-PET progressive disease was defined as ≥30% increase in SUV at either the primary site or lymph nodes involved pre-CRT, or if new hypermetabolic lesions occurred outside the original disease window. FDG-PET stable disease encompassed all values in between.

For patients who received trimodality therapy, pathologic examination of all surgical resection specimens was performed. Histopathological examination included descriptions of tumor type and extension, lymph node status, and resection margins. The degree of histopathological regression was graded according to our previously described five-tier method [22]. Using this method, specimens were classified as complete response (no residual tumor), microscopic residual (microscopic residual with questionable viability), partial response (downstaging of pretreatment TNM staging), stable disease (no change in staging), or progressive disease (upstaging).

Outcomes were defined from the date of diagnosis to the date of death or date of last follow-up. For comparisons by chemotherapy group, chi-squared tests and Fisher’s exact tests were performed for categorical variables, while t-tests were performed for normal continuous variables and Mann-Whitney U tests were performed for non-normal continuous and ordinal variables. Independent variables included sex, age, race, Eastern Cooperative Oncology Group (ECOG) performance status, stage, histology, tumor location, radiotherapy dose, weight loss during CRT, therapy regimen, radiographic response, and pathologic response. Overall survival (OS) and progression free survival (PFS) were estimated for the two chemotherapy groups in the bivariate setting using the Kaplan-Meier method, while proportional hazards models were used to estimate adjusted hazard ratios for chemotherapy group and key covariates (age, gender, ECOG score, stage, and surgical status). Statistical analysis was performed using R version 3.2.5 software (R Foundation for Statistical Computing, Vienna, Austria) and SAS version 9.4 software (SAS Institute Inc., Cary, NC, USA). A two-tailed alpha level of 0.05 was used throughout.

Hematologic toxicity data availability was limited in patients treated prior to 2012. Due to the low numbers of patients treated with cisplatin/5-FU in the post 2012 time period (n = 9), we utilized toxicity data from a trial investigating esophagectomy versus cisplatin/5-FU given concurrent with radiation followed by esophagectomy (CALGB 9781) [23]. Two by two contingency tables of observed rates of grade 3 and 4 toxicity in the carboplatin/paclitaxel group were generated from the present study and from corresponding event rates among patients treated with cisplatin/5-FU as reported in CALGB 9781. These rates were compared between groups using the Fisher’s exact test.

## Results

Patient characteristics

Of 364 patients, 261 patients were identified that had undergone treatment with one of the two chemotherapy regimens of interest, of whom 128 patients received cisplatin/5-FU and 133 patients received carboplatin/paclitaxel. Patient characteristics for these 261 patients can be found in Table 1, both for the entire cohort and stratified by chemotherapy regimen.

**Table 1:** Patient and treatment characteristics for the entire cohort and stratified by chemotherapy regimen. 5-FU = 5-fluorouracil; Adeno = adenocarcinoma; CRT = chemoradiotherapy; CT = computed tomography; ECOG = Eastern Cooperative Oncology Group performance status; IQR = interquartile range; M = metastasis, N = nodal; PET = positron emission tomography; RT = radiotherapy; SCCa = squamous cell carcinoma; T = tumor.

Patient Characteristics		Overall (n = 261)	Carboplatin/Paclitaxel (n = 133)	Cisplatin/5-FU (n = 128)	p
Sex (%)	Female	48 (18.4)	24 (18.0)	24 (18.8)	1.00
	Male	213 (81.6)	109 (82.0)	104 (81.2)	
Race (%)	Caucasian	231 (88.5)	122 (91.7)	109 (85.2)	0.34
	African American	26 (10.0)	10 (7.5)	16 (12.5)	
	Hispanic	3 (1.1)	1 (0.8)	2 (1.6)	
	Asian	1 (0.4)	0 (0.0)	1 (0.8)	
Age (mean (SD))		62.79 (10.67)	65.46 (10.00)	60.01 (10.67)	<0.01
ECOG (%)	0	82 (31.4)	32 (24.1)	50 (39.1)	0.1
	1	147 (56.3)	81 (60.9)	66 (51.6)	
	2	25 (9.6)	16 (12.0)	9 (7.0)	
	3	4 (1.5)	2 (1.5)	2 (1.6)	
	Missing	3 (1.1)	2 (1.5)	1 (0.8)	
T stage (AJCC 7^th^ ed.) (%)	0	20 (7.7)	15 (11.3)	5 (3.9)	0.01
	1	19 (7.3)	12 (9.0)	7 (5.5)	
	2	35 (13.4)	19 (14.3)	16 (12.5)	
	3	171 (65.5)	85 (63.9)	86 (67.2)	
	4	13 (5.0)	1 (0.8)	12 (9.4)	
	Missing	3 (1.1)	1 (0.8)	2 (1.6)	
N stage (AJCC 7^th^ ed.) (%)	0	83 (31.8)	48 (36.1)	35 (27.3)	<0.01
	1	145 (55.6)	59 (44.4)	86 (67.2)	
	2	29 (11.1)	25 (18.8)	4 (3.1)	
	3	2 (0.8)	1 (0.8)	1 (0.8)	
	Missing	2 (0.8)	0 (0.0)	2 (1.6)	
M stage (AJCC 7^th^ ed.) (%)	0	236 (90.4)	121 (91.0)	115 (89.8)	0.75
	1	22 (8.4)	10 (7.5)	12 (9.4)	
	Missing	3 (1.1)	2 (1.5)	1 (0.8)	
Stage (AJCC 7^th^ ed.) (%)	I	15 (5.7)	11 (8.3)	4 (3.1)	0.1
	II	89 (34.1)	51 (38.3)	38 (29.7)	
	III	130 (49.8)	60 (45.1)	70 (54.7)	
	IV	22 (8.4)	10 (7.5)	12 (9.4)	
	Missing	5 (1.9)	1 (0.8)	4 (3.1)	
Histology (%)	SCCa	59 (22.6)	28 (21.1)	31 (24.2)	0.69
	Adeno	199 (76.2)	104 (78.2)	95 (74.2)	
	Other	2 (0.8)	1 (0.8)	1 (0.8)	
	Missing	1 (0.4)	0 (0.0)	1 (0.8)	
Tumor Location (%)	Cervical	8 (3.1)	2 (1.5)	6 (4.7)	0.05
	Upper Thoracic	10 (3.8)	5 (3.8)	5 (3.9)	
	Middle Thoracic	32 (12.3)	16 (12.0)	16 (12.5)	
	Lower Thoracic	99 (37.9)	42 (31.6)	57 (44.5)	
	Gastroesophageal Junction	112 (42.9)	68 (51.1)	44 (34.4)	
Treatment Category (%)	Definitive CRT	142 (54.4)	75 (56.4)	67 (52.3)	0.81
	Trimodality	117 (44.8)	57 (42.9)	60 (46.9)	
	Other	2 (0.8)	1 (0.8)	1 (0.8)	
PET used for Imaging Response	No	118 (45.2)	78 (58.6)	40 (31.2)	<0.01
	Yes	143 (54.8)	55 (41.4)	88 (68.8)	
Primary Tumor Imaging Response (%)	Complete Response	42 (16.1)	19 (14.3)	23 (18.0)	0.56
	Partial Response	131 (50.2)	64 (48.1)	67 (52.3)	
	Stable Disease	51 (19.5)	31 (23.3)	20 (15.6)	
	Progressive Disease	13 (5.0)	6 (4.5)	7 (5.5)	
	Missing	24 (9.2)	13 (9.8)	11 (8.6)	
Regional Nodal Imaging Response (%)	Complete Response	123 (47.1)	64 (48.1)	59 (46.1)	0.26
	Partial Response	62 (23.8)	35 (26.3)	27 (21.1)	
	Stable Disease	20 (7.7)	11 (8.3)	9 (7.0)	
	Progressive Disease	31 (11.9)	10 (7.5)	21 (16.4)	
	Missing	25 (9.6)	13 (9.8)	12 (9.4)	
Post-operative Death (%)	No	101 (86.3)	50 (87.7)	51 (85.0)	0.35
	Yes	12 (10.3)	4 (7.0)	8 (13.3)	
	Missing	4 (3.4)	3 (5.3)	1 (1.7)	
Weight Pre-Tx (median [range])		172.00 [70.00, 306.00]	171.50 [92.00, 287.00]	173.00 [70.00, 306.00]	
Weight Post-Tx (median [range])		162.50 [65.00, 280.00]	166.00 [83.00, 277.00]	161.00 [65.00, 280.00]	
Weight Change (median [range])		-8.50 [-33.00, 28.00]	-7.00 [-29.00, 28.00]	-11.00 [-33.00, 16.00]	<0.01
Weight Change Percent (median [range])		-5.34 [-22.62, 24.74]	-4.06 [-16.56, 24.74]	-6.49 [-22.62, 10.06]	<0.01
Elapsed Days to Complete RT (median [range])		38.00 [6.00, 107.00]	39.00 [9.00, 57.00]	38.00 [6.00, 107.00]	0.06
RT Dose Prescribed (median [range])		5040.00 [740.00, 6300.00]	5040.00 [1460.00, 6000.00]	5040.00 [740.00, 6300.00]	

The mean age at diagnosis was 62.8 years (±10.7). Patients were predominantly male (81.6%), Caucasian (88.5%), and with adenocarcinoma histology (76.2%). Tumors were mainly located in the distal esophagus (37.9%) and gastroesophageal junction (42.9%) regions. The majority of patients (49.8%) had stage III disease at diagnosis.

The mean age at diagnosis was significantly higher for the carboplatin/paclitaxel group (65.5 years) compared to 60.0 years for the cisplatin/5-FU group (p < 0.01, Table 1). The carboplatin/paclitaxel group had slightly less advanced T-stage (p = 0.01) and slightly more advanced N-stage (p < 0.01) than the cisplatin/5-FU group. There was also a trend toward increased frequency of gastroesophageal junction tumors compared to distal tumors in the carboplatin/paclitaxel group. The remainder of the baseline clinical characteristics are described in Table 1.

Treatment response

PET/CT was acquired following CRT for response assessment in 143 patients (54.8%). Fifty-five (41.4%) patients receiving carboplatin/paclitaxel and 88 (68.8%) patients receiving cisplatin/5-FU had post-treatment PET/CT imaging (p < 0.01). There were no differences in PET/CT complete response at the primary site between carboplatin/paclitaxel (25.5%) and cisplatin/5-FU (23.9%) (p = 0.80) or in the regional nodes (69.1% versus 55.7%, respectively) (p = 0.32). For patients treated with trimodality, a pCR was seen in 25.7%, partial response was seen in 56.9%, and stable/progressive disease seen in 17.5% for the entire cohort. When stratified by chemotherapy, the pCR with carboplatin/paclitaxel vs. cisplatin/5-FU was 29.6% (16/54) vs. 21.8% (12/55), partial response was 64.8% (35/54) vs. 49.1% (27/55), and stable/progressive disease was 5.6% (3/54) vs. 29.1% (16/55), p = 0.03 (Table 2).

**Table 2:** Pathologic response for trimodality patients by chemotherapy regimen. 5-FU = 5-fluorouracil.

Pathologic Response	Overall (n = 109) (%)	Carboplatin/Paclitaxel (n = 54) (%)	Cisplatin/5-FU (n = 55) (%)	p
Complete Response	28 (25.7)	16 (29.6)	12 (21.8)	0.03
Partial Response (microscopic residual with questionable viability)	24 (22.0)	13 (24.1)	11 (20.0)	
Partial Response	38 (34.9)	22 (40.7)	16 (29.1)	
Stable	10 (9.2)	2 (3.7)	8 (14.5)	
Progressive	9 (8.3)	1 (1.9)	8 (14.5)	

Survival outcomes

**Figure 1:**
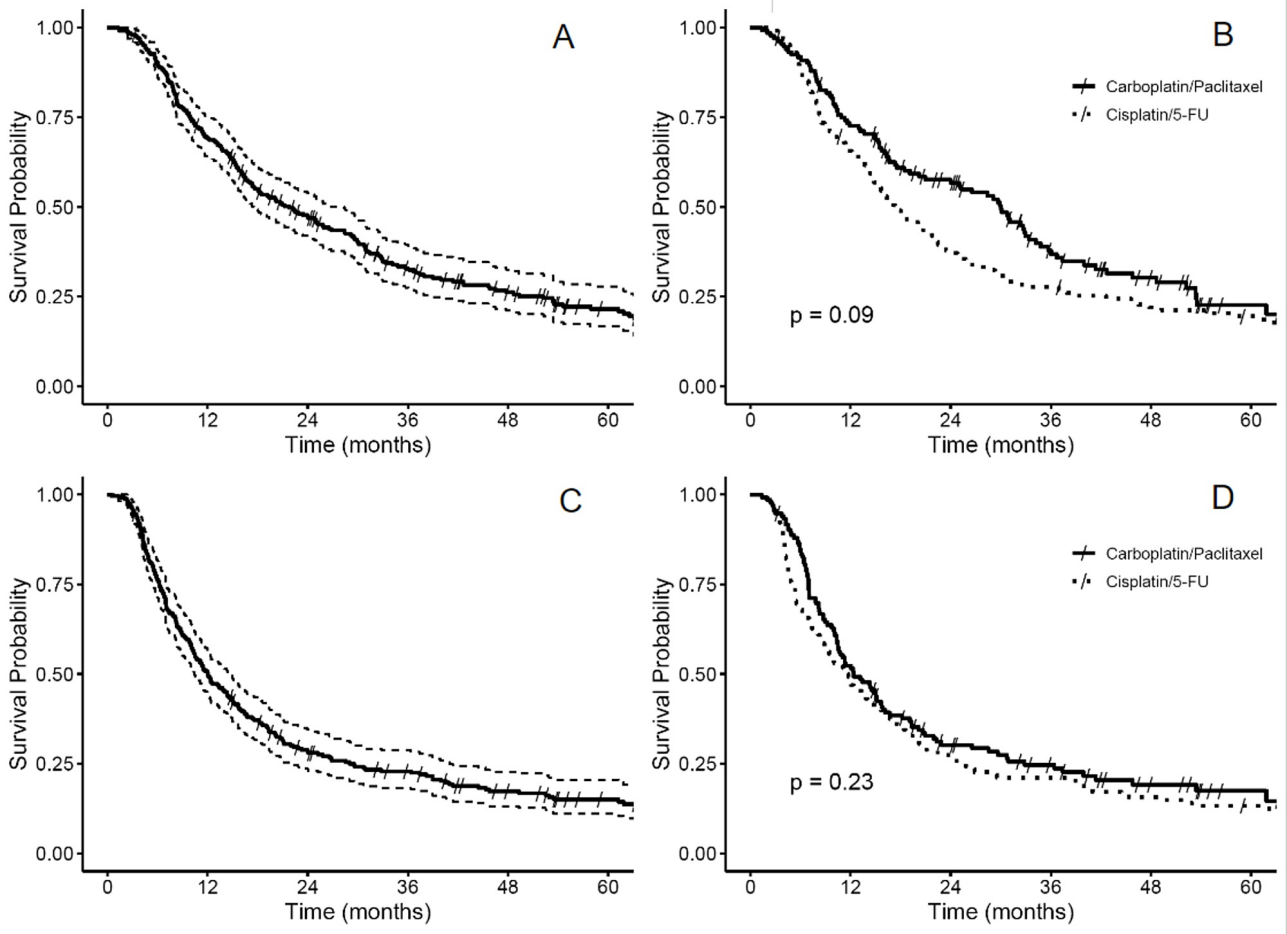
Kaplan-Meier analyses of OS for all patients (A) and as stratified by chemotherapy (B). Kaplan-Meier analyses of PFS for all patients (C) and as stratified by chemotherapy (D). OS = overall survival; PFS = progression free survival.

For the entire cohort, the median follow-up was 16.2 months (range: 1.0-235.2) and 42.2 months (range: 3.2-235.2) for surviving patients. Median OS was 21.8 months (95% CI: 17.5-28.4) and median PFS was 12.0 months (95% CI: 10.4-14.5) (Figure 1).

Median OS was 30.0 months (95% CI: 21.0-34.2) with carboplatin/paclitaxel and 17.3 months (95% CI: 14.6-22.4) with cisplatin/5-FU (p = 0.09). We found no significant effect modification of this overall association between chemotherapy regimen and OS, which was statistically non-significant, but directionally in favor of carboplatin/paclitaxel after stratifying by trimodality status. Among those treated with trimodality therapy, median OS was 32.4 months (95% CI: 29.6-53.4) for those treated with carboplatin/paclitaxel vs. 25.8 months (95% CI: 19.3-45.7) for those treated with cisplatin/5-FU (log-rank p-value = 0.62). For patients treated with CRT alone, median OS was 20.2 months (95% CI: 15.5-33.4) with carboplatin/paclitaxel and 13.5 months (95% CI: 10.7-18.0) with cisplatin/5-FU (log-rank p = 0.05).

Median PFS was 12.4 months (95% CI: 10.5-15.9) with carboplatin/paclitaxel and 11.6 months (95% CI: 8.9-15.7) with cisplatin/5-FU (log-rank p = 0.23). Again, we found no effect modification of the overall chemotherapy and PFS association after stratification by trimodality status. Among those treated with trimodality therapy, median PFS was 14.9 months (95% CI: 11.4-26.5) for carboplatin/paclitaxel and 18.4 months (95% CI: 13.8-25.2) for cisplatin/5-FU (log-rank p = 0.97). Kaplan-Meier estimates of OS and PFS for all patients and as stratified by chemotherapy regimen can be found in Figure 1.

Because there was no significant effect modification by trimodality status in the above survival analyses, we performed proportional hazards models on the total sample, although we adjusted for surgical status (definitive CRT vs. trimodality). We also adjusted for age (continuous, in years), gender, and ECOG score, grade, and stage (all ordinal). In the multivariable proportional hazards model of OS (Table 3), the adjusted hazard ratio (HR) comparing carboplatin/paclitaxel to cisplatin/5-FU was 0.75 (95% CI: 0.55-1.03, p = 0.08).

**Table 3:** Multivariable proportional hazards model of overall survival. Cis = Cisplatin; 5-FU = 5-fluorouracil; ECOG = Eastern Cooperative Oncology Group performance status; CRT = Chemoradiotherapy.

	Hazard Ratio	95% CI	p
Carboplatin/Paclitaxel (versus Cisplatin/5-FU)	0.75	0.55-1.03	0.08
Age (years)	1.00	0.99-1.02	0.74
Female (versus male)	0.64	0.40-1.03	0.06
ECOG 2-3 (versus 0-1)	1.09	0.66-1.81	0.73
Stage	1.28	1.02-1.59	0.03
Grade	1.31	0.99-1.71	0.05
Trimodality Therapy (versus definitive CRT)	0.48	0.34-0.69	<0.01

Factors that were found to be statistically significant independent predictors of overall survival were stage and trimodality therapy (versus definitive CRT) with HR of 1.28 (95% CI: 1.02-1.59, p = 0.03) and 0.48 (95% CI: 0.34-0.69, p < 0.01), respectively. For PFS (Table 4), there was no significant effect of carboplatin/paclitaxel compared to cisplatin/5-FU when adjusting for the same set of variables (HR = 0.84, p = 0.27).

**Table 4:** Multivariable proportional hazards model of progression-free survival. 5-FU = 5-fluorouracil; CRT = chemoradiotherapy; ECOG = Eastern Cooperative Oncology Group performance status.

	Hazard Ratio	95% CI	p
Carboplatin/Paclitaxel (versus Cisplatin/5-FU)	0.84	0.62-1.15	0.27
Age (years)	0.99	0.98-1.01	0.86
Female (versus male)	0.61	0.39-0.95	0.03
ECOG 2-3 (versus 0-1)	1.01	0.63-1.62	0.97
Stage	1.39	1.11-1.72	<0.01
Grade	1.44	1.11-1.85	<0.01
Trimodality Therapy (versus definitive CRT)	0.46	0.33-0.65	<0.01

Stage (HR 1.39, p < 0.01), grade (HR 1.44, p < 0.01), female gender (HR 0.61, p = 0.03), and trimodality therapy (HR 0.46, p < 0.01) were significantly associated with PFS.

Toxicity

**Figure 2:**
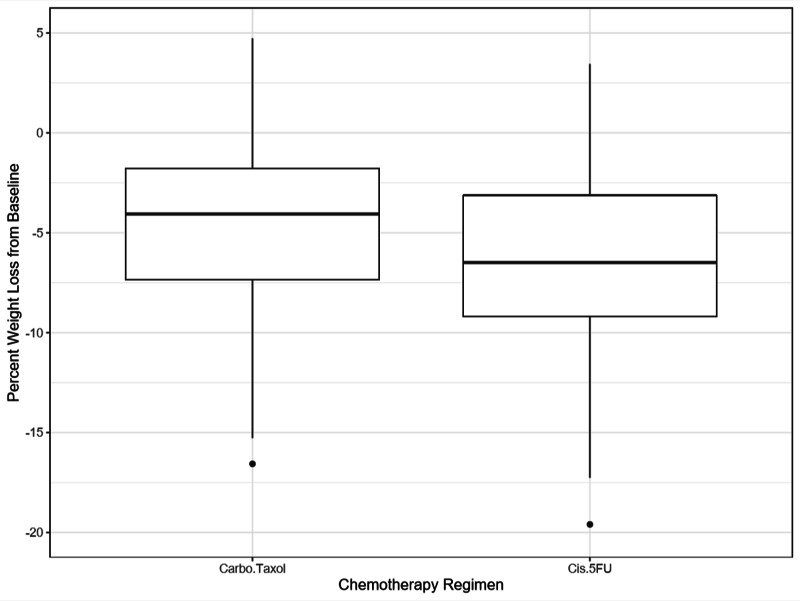
Weight change from baseline following completion of chemoradiotherapy for the entire cohort. 5-FU = 5-fluorouracil.

For all patients, weight loss during chemoradiotherapy was lower in patients receiving carboplatin/paclitaxel (median: 7.0 pounds; 4.1% body weight) vs. cisplatin/5-FU (median: 11.0 pounds; 6.5% body weight) (p < 0.01). Box plots demonstrating weight change by chemotherapy regimen can be found in Figure 2.

There was no difference in 30-day postsurgical mortality between the two chemotherapy regimens (7.0% versus 13.3%, respectively) (p = 0.35). There were 126 patients treated from 2012 through 2018, 103 of which were treated with carboplatin/paclitaxel and had laboratory data available. Hematologic grade 3/4 toxicity rates were: leukopenia 30.3%, neutropenia 16.8%, lymphocytopenia 80.7%, anemia 7.6%, and thrombocytopenia 5%. Comparison of a cohort of patients treated with cisplatin/5-FU in CALGB 9781 found an increase in grade 3 lymphocytopenia with carboplatin/paclitaxel but no difference in the rate of grade 4 lymphocytopenia (Table 5).

**Table 5:** Hematologic toxicity in the carboplatin/paclitaxel group compared with hematologic toxicity reported from the CALGB 9781 clinical trial. All data are reported as count (frequency). *Indicates significant difference (Fisher’s exact p < 0.001).

	Carboplatin/Paclitaxel (Present Study)			Cisplatin/5-FU (CALGB 9781)		
	Grade 3	Grade 4	Number of Patients Reporting	Grade 3	Grade 4	Number of Patients Reporting
WBC	30 (29)	6 (6)	103	7 (25)	3 (11)	28
Granulocytes	17 (17)	3 (3)	98	4 (15)	3 (12)	26
Lymphocytes	61 (59)*	35 (34)	103	2 (8)*	10 (38)	26
Hemoglobin	6 (6)	3 (3)	103	3 (11)	1 (4)	28
Platelets	6 (6)	0 (0)	103	2 (7)	1 (4)	27

## Discussion

The optimal treatment regimen for patients with advanced esophageal cancer remains controversial. The use of trimodality therapy has been shown to significantly improve patient outcomes in the treatment of esophageal cancer compared to surgery alone [2,4,9]. However, which chemotherapy regimen is superior for optimizing clinical outcomes and survival remains in question. While concurrent use of cisplatin/5-FU has been a leading standard regimen since the 1980s, the publication of the CROSS trial in 2012 led to a widespread transition to the concurrent use of carboplatin/paclitaxel due to the favorable toxicity profiles and improved survival reported with its use [9].

In this study of 261 patients with advanced esophageal cancer undergoing CRT with or without surgical resection, we examine two standard chemotherapy regimens, given concomitantly with irradiation, used in the treatment of esophageal cancer to evaluate if differences exist between them in optimizing patient response to therapy, outcomes, and survival. The surgical and radiation approaches did not significantly vary during this study. The initial study regarding the use of carboplatin and paclitaxel in esophageal cancer was a phase I study involving patients with metastatic esophageal cancer [24]. There has not been a large phase III study which directly compares these two chemotherapy regimens. The literature comparing the two regimens is limited and includes multiple small retrospective studies [25-27]. This current study represents one of the largest published series we are aware of which directly evaluates cisplatin/5-FU versus carboplatin/paclitaxel for the treatment of esophageal cancer in the definitive CRT and neoadjuvant setting.

We have found that the widely utilized CROSS trial regimen of concurrent chemotherapy with carboplatin/paclitaxel is associated with lower toxicity as defined by weight loss during treatment when compared to cisplatin/5-FU. The lower toxicity profile associated with the systemic use of carboplatin/paclitaxel has already been well documented in the literature [15,19]. We did find a numerical but non-significant trend toward increased post-operative deaths for patients receiving cisplatin/5-FU. Unfortunately our electronic medical record changed in 2012 which limited direct comparison of toxicity between the two regimens as the majority of patients who received cisplatin/5-FU were treated prior to 2012. In order to provide some form of comparison in regards to hematologic toxicity we examined the rates of hematologic toxicity that were reported in patients undergoing the CALGB 9781 study which was investigating patients randomized to esophagectomy alone vs. chemoradiation with cisplatin/5-FU followed by esophagectomy. Hematologic toxicity data in our patients that underwent treatment with carboplatin/paclitaxel showed similar rates of grade 3/4 toxicity when comparing to those patients that underwent treatment with cisplatin/5-FU in CALGB 9781 [23]. Interestingly, the reported grade 3 lymphocytopenia in the CALGB 9781 study was lower than the number having grade 4 toxicity, which is not the typical pattern in terms of rate and grade of toxicity. This is likely explained from the low numbers of patients accrued in the trial and a bias in the patients that had toxicity data available. Overall, our findings suggest somewhat decreased toxicity with carboplatin/paclitaxel as compared to cisplatin/5-FU which is similar to what has been reported in the literature in the studies comparing these two regimens [25-27].

The perception that the combination of carboplatin/paclitaxel is a relatively less aggressive regimen compared to cisplatin/5-FU has also led many critics to question its efficacy for improving pathologic response and survival. In this study, our finding of an improvement in both pathologic response for trimodality patients and a trend for improvement in overall survival for the entire cohort when patients were treated with carboplatin/paclitaxel compared to patients treated with cisplatin/5-FU appears to contradict this. Several previous studies have linked pathologic response to CRT with clinical outcomes [28,29], but these studies only involve patients treated with a cisplatin-based chemotherapy regimen. Even with improvement in pathologic response in the use of carboplatin/paclitaxel, no significant correlation between chemotherapy regimen and PFS or OS was observed. There was an improvement in OS in patients with lower stage and those able to undergo trimodality therapy compared to those receiving just definitive CRT. Both of these findings are expected as patients with worse disease burden and those unable to undergo surgical resection due to comorbidities or performance status would likely have worse outcomes.

Our results should be interpreted with caution given the retrospective nature of this study. It is prone to patient selection bias with differences in surgical operability as well as loss of patient follow-up due to frequent death and comorbid illnesses in this population of patients with esophageal cancer. The toxicity profile differences between the two chemotherapy regimens have been described in other studies and our study was limited in direct comparison of toxicity differences between the two regimens and particularly limited to assessment of change in patient weight and hematologic grading. These limitations can be explained by the long span of time included in this study as well as the varying degree of reporting and different electronic medical records used by our institution over the study time period. While we are not aware of any current prospective trials comparing the two chemotherapy regimens used in the study, the ongoing randomized Phase II PROTECT trial is directly comparing radiation with either carboplatin/paclitaxel or with FOLFOX chemotherapy and is one study that is varying chemotherapy regimens in an effort to improve outcomes in patients with esophageal cancer [30]. Further research is vital for continuing to improve treatment strategies for patients with esophageal carcinoma.

## Conclusions

In conclusion, we present one of the largest published series directly evaluating cisplatin/5-FU versus carboplatin/paclitaxel for the treatment of esophageal cancer in the definitive chemoradiation and neoadjuvant setting. We have provided evidence that the use of carboplatin/paclitaxel is the desired alternative to cisplatin/5-FU in both of these settings. There appears to be an improved tolerability in terms of weight loss and cytopenias in conjunction with evidence for an improvement in pathologic response with the use of carboplatin/paclitaxel.
